# Prediction of Anthocyanidins Content in Purple Chinese Cabbage Based on Visible/Near Infrared Spectroscopy

**DOI:** 10.3390/foods12091922

**Published:** 2023-05-08

**Authors:** Ya-Qin Wang, Guang-Min Liu, Li-Ping Hu, Xue-Zhi Zhao, De-Shuang Zhang, Hong-Ju He

**Affiliations:** 1 Institute of Agri-Food Processing and Nutrition, Beijing Academy of Agriculture and Forestry Sciences, Beijing 100097, China; wangyaqin@iapn.org.cn (Y.-Q.W.);; 2 Key Laboratory of Vegetable Postharvest Processing of Ministry of Agriculture and Rural Areas, Beijing 100097, China; 3 Beijing Vegetable Research Center, Beijing Academy of Agriculture and Forestry Sciences, Beijing 100097, China

**Keywords:** near infrared spectroscopy, vegetables, anthocyanidins, fast determination

## Abstract

Purple Chinese cabbage (PCC) has become a new breeding trend due to its attractive color and high nutritional quality since it contains abundant anthocyanidins. With the aim of rapid evaluation of PCC anthocyanidins contents and screening of breeding materials, a fast quantitative detection method for anthocyanidins in PCC was established using Near Infrared Spectroscopy (NIR). The PCC samples were scanned by NIR, and the spectral data combined with the chemometric results of anthocyanidins contents obtained by high-performance liquid chromatography were processed to establish the prediction models. The content of cyanidin varied from 93.5 mg/kg to 12,802.4 mg/kg in PCC, while the other anthocyanidins were much lower. The developed NIR prediction models on the basis of partial least square regression with the preprocessing of no-scattering mode and the first-order derivative showed the best prediction performance: for cyanidin, the external correlation coefficient (RSQ) and standard error of cross-validation (SECV) of the calibration set were 0.965 and 693.004, respectively; for total anthocyanidins, the RSQ and SECV of the calibration set were 0.966 and 685.994, respectively. The established models were effective, and this NIR method, with the advantages of timesaving and convenience, could be applied in purple vegetable breeding practice.

## 1. Introduction

Chinese cabbage (*Brassica rapa* L. ssp. *pekinensis*) is the most widely cultivated and consumed vegetable in East Asia with the characteristics of high yield, good cold resistance, long supply period, and rich nutrition. The inner leaf color of Chinese cabbage is mainly white and yellow. Purple leaf Chinese cabbage (PCC) is mainly generated by the cross of common green Chinese cabbage with red leaf mustard (*Brassica juncea* Coss.), purple flowering Chinese cabbage, or red bok choy (*Brassica rapa* L. ssp. *chinensis*) [1,2]. It has become increasingly popular due to its beautiful color, special flavor, and high level of anthocyanidins [2]. Anthocyanidins, a class of flavonoid substances, exist in different colors in fruits, flowers, and vegetables, such as purple, blue, and red. They contain a C6-C3-C6 carbon skeleton and -OH or -OCH_3_ groups and specific sugar or acylated sugar residues located at C3, C5, and C7 positions [3,4]. Based on the type and location of the substituents, anthocyanidins are generally classified into six major groups: cyanidin, delphinidin, petunidin, malvidin, peonidin and pelargonidin, and the main anthocyanidin in PCC is cyanidin accumulated in the vacuoles [2,3,5]. Anthocyanidins have a wide range of anti-inflammatory, cardioprotective, chemotherapy, and hepatoprotective effect for human disease prevention [6]. Studies have proved that anthocyanidins have a good role in the chemoprevention and treatment of breast cancer [7]. Blueberry anthocyanidins can effectively improve the solubility of lipids [8], and extracted anthocyanidins from apples have an inhibitory effect on gastric cancer cells [9]. Due to their human health benefits, anthocyanidins have received more and more attention from public in recent years [9,10]. Creating colorful leaf vegetables, such as PCC, which contain abundant anthocyanidins, is of significant commercial interest and the new trend of breeding.

Visible/near-infrared spectroscopy (NIR) is a widely used technique in the agriculture and food industry with the advantages of fast, non-destructive, environmentally friendly, and accurate analysis. NIR is a molecular vibrational spectrum with wavelengths ranging from 400–750 (visible) and 750–2500 nm (near-infrared), in which the absorption signals of the reflected chemical components are assigned mainly to overtone and octave vibrations of hydrogen-containing groups, including C-H, N-H, O-H, and S-H [11,12]. Theoretically, no two compounds produce the same visible/near-infrared spectra since their unique composition of atoms [12]. It has been widely used in the field of bioactive compound detection in vegetables and fruits, and its applicability has been proven. Prodromidis et al. have successfully used FT-IR and UV-Vis spectroscopy to measure the onion anthocyanidins during heating [13]. Johnson et al. used attenuated total reflection Fourier transform infrared spectroscopy to predict the total anthocyanidin content in ethanolic extracts of plum with an R^2^ of 0.93 [14]. Additionally, using NIR spectroscopy in the prediction of anthocyanidins content and antioxidant activity in grape juice is feasible [15]. Tian et al. established a prediction model for the detection of water content and anthocyanidins content in purple potatoes by visible near-infrared hyperspectroscopy [16]. With the development of algorithms, chemometrics, and artificial intelligence, the application of NIR spectroscopy will be extended for fast screening and quantitative analysis of anthocyanidins.

Purple leaf Chinese cabbage has become a popular breeding interest; meanwhile fast and accurate determination of the anthocyanidins contents is an important task for improving its nutritional quality. The commonly used determination methods for anthocyanidins are based on ultrasonic or microwave-assisted liquid extractions and high-performance liquid chromatography (HPLC) and liquid chromatography-tandem mass spectrometry (LC-MS/MS) detection [17,18]. However, the extraction is complicated and time-consuming while the reagents used may be harmful to the environment and human health [19], and the equipment are more expensive and require experts for analysis. By comparing with chemical analysis techniques, spectroscopic techniques are relatively simple and do not require further expansion of sample preparation [20]. NIR spectroscopy could be a powerful tool to fulfill this task. To date, no studies have focused on the quantitative prediction of anthocyanidins in PCC by NIR spectroscopy. It is an urgent need to build a suitable NIR method for simple and fast prediction of anthocyanidins to help the breeders and producers since the prediction models established by different food matrices cannot be simply applied to PCC. Therefore, this study aims to develop an accurate quantitative prediction method for anthocyanidin content in PCC using NIR spectroscopy, which laid a foundation for the fast and convenient detection of the nutritional quality of agri-food and the rapid screening of purple vegetable breeding materials.

## 2. Materials and Methods

### 2.1. Sample Preparation

The purple leaf Chinese cabbage samples from different breeding backgrounds with distinct color phenotypes were collected from Beijing Vegetable Research Center (Beijing, China, 116°30′ E, 39°94′ N). Specifically, the purple color trait was from the variety of 15NG28, as previously described [21], and the green parents were different Chinese cabbages with distinct shapes of leaves, holding patterns, and maturity traits. Totally 106 PCC samples were harvested and transferred to the laboratory within half an hour on 19 November 2021. Then, the vegetable leaves of each sample were cut into 2.0 cm length pieces, uniformly mixed, and freeze-dried (BIOCOOL vacuum freeze dryer, Boyikang Co., Ltd., Beijing, China). The dry samples were ground into a fine powder, passed through an 80-mesh sieve, then stored at −40 °C for further anthocyanidins content determination and NIR spectral profiles acquisition.

### 2.2. HPLC Analysis of Anthocyanidins

Extraction and HPLC analysis of anthocyanidins in PCC were carried out according to the Agricultural Industry Standard of the People’s Republic of China (NY/T 2640-2014, Determination of anthocyanidins in plant origin products-High performance liquid chromatography). Basically, accurately weighed 0.200 g powdered samples were placed in a 15 mL plastic tube, and 5.00 mL of extracting solution consisting of ethanol:water:hydrochloric acid = 2:1:1 (volume) was added to extract anthocyanidins. The extraction mixture was sonicated for 30 min at room temperature, then hydrolyzed under boiled water for one hour. Then, the cooled extraction mixture was centrifuged using a HITACHI high-speed refrigerated centrifuge (Katsuta, Japan) at 8000 rpm for 10 min. The supernatant was accurately fixed to 5.00 mL volume and filtered through a 0.45 μm polyvinylidene fluoride syringe filter before HPLC analysis.

The quantification of anthocyanidins was carried out on a reversed-phase HPLC system (LC-20AD, Shimadzu, Tokyo, Japan) coupled with a photodiode array (PDA) detector (SPD-M20A, Shimadzu, Tokyo, Japan). The column used was a Waters C18 (3.9 × 150 mm, 5 μm) kept at 35 °C. The gradient elution was carried out with a binary solvent system consisting of ultrapure water (A) and acetonitrile (B), both containing 1% formic acid, at a constant flow rate of 0.8 mL/min. The injection volume was 20 μL. Anthocyanidin compounds were detected at the wavelength of 530 nm. Individual anthocyanidins were quantified via comparison of the peak areas with those of the known standards. The anthocyanidins standards (delphinidin, cyanidin, petunidin, pelargonidin, peonidin, and malvidin) were purchased from Sigma-Aldrich (Darmstadt, Germany).

### 2.3. NIR Spectral Acquisition

The NIR spectrometer used in this study was a FOSS NIR Systems model 5000 (Foss NIRSystems Inc., Silver Spring, MD, USA). The NIR spectrometer was preheated for 30 min before the sample scanning, and the samples were only scanned when the spectra and noise tests were passed. The dried PCC powders were evenly spread in the sample round cups, respectively and compacted with the lid to ensure the sample powder was covered evenly. The spectra were scanned in the wavelength range of 400–1100 nm and 1100–2498 nm under diffuse reflection mode. Each sample was scanned three times. The scanned spectral curves were collected, and the data were processed using the Foss WinISI III calibration software throughout the whole process.

### 2.4. Data Processing

The PCC samples were divided into two sets by systematic sampling method; 86 of them were used as calibration sets to establish the prediction models, and 20 samples not involved in the calibration were used as validation sets for external validation of the effectiveness of the developed models. The chemical determination results of anthocyanidin content obtained by HPLC of the calibration set samples were imported into the chemometric software accompanying the instrument and processed for NIR spectroscopy to obtain a cal. file. The spectral data were preprocessed using a partial least squares regression (PLSR) method at three different wavelength bands. These three bands included 400–1100 nm, 1100–2498 nm (full band); 400–800 nm (visible band); 800–1100 nm, 1100–2498 nm (near infrared band). The pre-processing scattering model of the spectral data included no scattering processing (None), standard normal variables transformation + de-trending processing (SNV+Detrend), standard normal variation processing (SNV Only), de-trending processing (Detrend Only), standard multivariate scattering correction (Standard MSC), weighted multivariate scattering correction (Weighted MSC), and two different derivative treatments, namely, no derivative and first-order derivative were employed. The final prediction models built under different preprocessing methods were compared, and the model with the internal cross-validation correlation coefficient (1-VR) close to 1 and lower standard error of cross-validation (SECV) was selected as the best one. These two sets of data can basically reflect the prediction performance of the calibration model for unknown samples. Subsequently, samples of the validation set were analyzed to test the predictive ability of the proposed model. The criterion was that the higher the external correlation coefficient (RSQ) value and the lower the standard deviation of prediction (SEP), the more accurate the model.

## 3. Results

### 3.1. Anthocyanidins Contents in PCC Samples

Anthocyanidins contents of the PCC samples were analyzed by the HPLC method (the results are shown in Table S1), and the content distribution of the anthocyanidins fractions is shown in Table 1. Four kinds of anthocyanidins were detected, with cyanidin the most abundant one in PCC, which was a coincidence with the previous report [2]. Cyanindin had the largest range of content variation from 93.5 to 12,802.4 mg/kg, and the average content was 5741.2 mg/kg. In most samples, cyanidin accounted for more than 95% of the total anthocyanidins. The content distribution range of cyanidin in the selected samples was wide, which can well represent PCC samples with different contents of anthocyanidins; it meant that it was a suitable sample set for establishing of NIR model.

Delphinidin was detected in most PCC samples, with a content up to 193.7 mg/kg, and the average content was 159.4 mg/kg. Compared to cyaniding, pelargonidin and peonidin were much lower in PCC, whose average contents were 52.3 mg/kg and 45.5 mg/kg, respectively, accounted for less than 1% content to the total anthocyanidins in PCC.

### 3.2. Visible/NIR Spectral Analysis of PCC Samples

**Figure 1:**
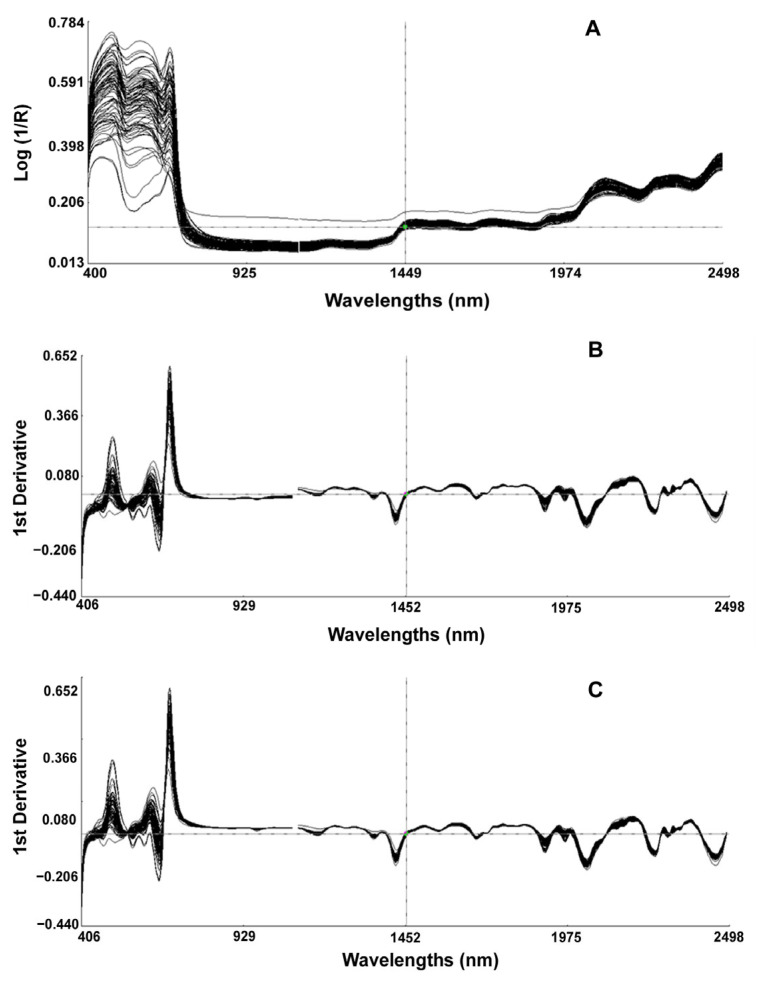
Visible/Near infrared spectra of purple leaf Chinese cabbages. (**A**): original spectra; (**B**): spectrum after SNV+Detrend and first derivative processing; (**C**) spectrum after SNV only and first derivative processing.

Using the software WinISS III, the chemically determined values were input to the corresponding spectral positions, and the spectral data were analyzed in combination with chemical analysis data. The raw spectra of the PCC samples obtained after visible/NIR spectroscopy scan (Figure 1A), in which the horizontal coordinate was the wavelength, and the vertical coordinate was the absorbance expressed as log 1/R, showed that several samples of PCC had a clear trend of decreasing absorption peaks in the wavelength range of 400 to 800 nm, which indicated that different samples had specific absorption characteristics in the visible wavelength band. The large variation in their spectrograms also indirectly indicated the different contents of each sample composition.

The raw NIR spectra contained comprehensive information on all chemical structures and a lot of irrelevant information and noise, so mathematical data pretreatment methods were applied to remove noise, compensate for baseline shifting, reduce the influence of non-target variation, and assist in smoothing the spectrum. The derivative transformation could partially compensate for baseline offset between samples and reduce instrument drift effects [22]. Figure 1B shows the spectral curve of the original spectrum after SNV+Detrend and first-order derivative pretreatment. The pretreated spectrum had more obvious undulations, the peaks became more and sharper, and the absorption peaks appeared in the originally smooth part. Figure 1C shows the spectral profile of the original spectrum after the SNV only and first-order derivative pretreatment, and the fitting phenomenon could be observed. On the processed spectrograms, we observed more clearly several characteristic peaks of the spectrum, with the peak at 672 nm associated with chlorophyll [23]. The peak at about 760 nm corresponds to the third overtone of the O-H vibration [24].

### 3.3. Establishment of Quantitative Models for Anthocyanidins Content in PPC

#### 3.3.1. Model for Cyanidin Content Prediction

**Figure 2:**
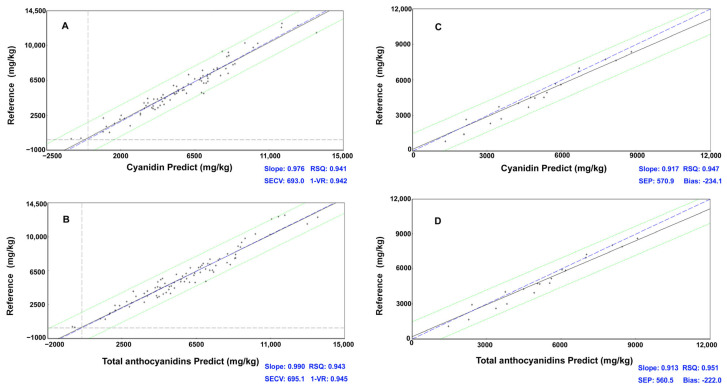
The cross-validation and external validation results of cyanidin and total anthocyanidin prediction models. (**A**): cross-validation of cyanidin prediction model; (**B**): cross-validation of total anthocyanidins prediction model; (**C**): external validation of cyanidin prediction model; (**D**): external validation of total anthocyanidins prediction model.

The spectral curves obtained from the scanned samples and the chemical analysis data were processed using PLSR to establish calibration models, and the calibration equation results are shown in Table 2. All spectral pre-treatment models performed well, with RSQ all above 0.91. Successful calibrations usually had a correlation coefficient of determination above 0.9. The 1-VR value of cyanidin in the full spectral band from 400 to 1100 nm and 1100 to 2498 nm after no scattering processing and first-order derivative pretreatment was 0.942 at the maximum, the SECV value was 693.004 at the smaller value, and the RSQ was 0.965. Figure 2A shows the cross-validation result of the prediction model established, the linear regression relationships between the NIR predicted values, and the chemically determined results (reference value). The slope of the line was 0.976, which is closed to 1; the samples were irregularly distributed on both sides of the line with the overall trend of discrete. The model fits well and can achieve the purpose of good quantitative prediction. So, the model after no scattering processing (None) and first-order derivative pretreatment was chosen to be used in the rapid screening of high-quality PCC breeding materials. The highest 1-VR of delphinidin prediction models was 0.172, and the SECV was 12.030, obtained by SNV only (first-order derivative) in the 400–800 nm band. The values of correlation coefficients were small and could not accurately predict the content of delphinidin fraction in PCC. After no scattering processing and first-order derivative preprocessing in 400–800 nm visible light of pelargonidin, the 1-VR value was 0.467 at maximum, and SECV value was 3.887 at minimum, so its detection model was poorly predictive and could not accurately predict the content of pelargonidin. After the Detrend only and first-order derivative pretreatment under 400–800 nm visible light, the 1-VR value was 0.652 at maximum, and the SECV value was 3.557 for peonidin, so its detection model prediction was weakly correlated and could not accurately predict the content of peonidin fraction in PCC, which need further study. Considering that the contents of delphinidin, pelargonidin, and peonidin were relatively low, which accounted for less than 5% of the total anthocyanidins, it is negligible of their contribution to the quality of PCC.

#### 3.3.2. Model for Total Anthocyanidins Content Prediction

The performances of total anthocyanidin content prediction models were parallel with the models for cyanidin content prediction because cyaniding was the vast majority of anthocyanidin in PCC. As shown in Table 3, the 1-VR value of the total anthocyanidins in the visible/NIR spectral bands from 400 to 1100 nm and 1100 to 2498 nm after no scattering processing and first-order derivative preprocessing was 0.944, the minimum SECV value was 685.994, and the external correlation coefficient RSQ was 0.968, which meant that the cross-test effect was very satisfying. Combined with the cross-validation result shown in Figure 2B, the line slope was 0.990, and the samples scattered with no big deviation. The content of total anthocyanidins in PCC can be accurately predicted using the model after pretreatment of no scattering processing and first-order derivative.

#### 3.3.3. External Validation of the Calibration Models

Using the mathematical model developed by WinISI III software, the samples not involved in the calibration were analyzed for external validation of the effectiveness of the developed model. The effectiveness of the validation was indicated by RSQ, SEP, and Bias. After validation, the RSQ were 0.947 and 0.951, respectively, for cyanindin and total anthocyanidins models at 400 to 1100 nm and 1100 to 2498 nm visible/NIR spectra, after no scattering processing and first-order derivative pretreatment (Figure 2C,D). The test deviation biases were small, which were −234.079 and −222.0, respectively. The slopes of the external validation prediction plots (the linear regression between the NIR predicted values and the chemically determined results) were 0.917 for cyanidin and 0.913 for total anthocyanidins. The validation samples were irregularly distributed on both sides of the line, and there was no big deviation, which meant that the models worked well; they could output accurate results for efficient and rapid screening of high anthocyanidins content materials. In addition, the validation results of delphinidin, pelargonidin, and peonidin prediction models showed very poor performance, as we could expect.

## 4. Discussion

As an osmoregulatory substance, anthocyanins are one of the most important pigments in plant leaves. It has an irreplaceable role in improving the cold, drought, and disease resistance of plants, and, therefore, monitoring the content of anthocyanidins in plants can help to understand the physiological state of plants [3]. Meanwhile, the benefits for human health of anthocyanidins have drawn a great deal of people’s attention. At present, the detection of anthocyanidins contents in plants and plant products mainly uses HPLC or HPLC-MS method; the application of rapid and non-destructive detection using NIR is still in its initial stage, but high throughput, convenient operation, and no use of organic solvents will make NIR a powerful support tool in horticulture practice and agri-food industry. Huang et al. [25] proposed a NIR spectroscopic detection method based on an ant colony algorithm (ACO) combined with interval partial least squares (iPLS) in order to detect anthocyanidins content in flower tea quickly and accurately, indicating that NIR spectroscopy has promising applications in measuring total anthocyanidins in plants. NIR spectroscopy can be used to determine the anthocyanidin content of berries in completely satisfied results without breaking the composition of the berries [26]. In this study, we successfully developed suitable prediction models for cyanidin and total anthocyanin content in PCC, and they could be applied in the breeding practice of PCC to realize rapid and efficient screening of high-quality breeding materials.

NIR spectroscopy belongs to an indirect analysis technology; the accuracy of the prediction result relies on the quality of the calibration models. So, the establishment of a high-quality model, with accurate chemical analysis and spectrum scanning data, strong anti-interference capability, and broad enough representation, is vitally important. A large number and representative sample sets are essential factors for model building. Additionally, an appropriate algorithm to divide sample subsets is also critical [27]. In order to expand the application scope of our established models, further improvement using a larger number of PCC samples with different breeding backgrounds and distinct phenotypes is required. In terms of algorithms for NIR model establishment, there are several regression methods frequently used for the prediction/quantification of chemical content, including multiple linear regression (MLR), principal component analysis (PCA) for the exploration of the data, and partial least squares regression (PLSR) analysis to obtain a quantitative prediction of the parameters of interest [12,28]. Among them, PLSR is the most widely used multivariate statistical data analysis method for quantitative analysis of the NIR spectrum, with strong anti-interference ability. In this study, we used a PLSR method to process the spectral data, and the quantitative prediction results were satisfied.

Compared to chemical analysis methods, the sensitivity of NIR spectroscopy is relatively low and cannot be used for trace analysis, but its modeling is suitable for the detection of components with high content and a wide range of variation. In a previous report, a satisfied NIR prediction model was established to detect anthocyanidin content in flower teas with a content range of 0.17 to 1.60 mg/g [25]. In this study, the model prediction performance of cyanidin content and total anthocyanidin content with a wide range of variation was relatively good, which could be used for rapid screening of breeding materials and prediction of anthocyanidin content in PCC breeding practice. Meanwhile, the prediction model performance of delphinidin, pelargonidin, and petunidin with less abundant contents in PCC was very poor. Considering the contents of these three anthocyanidins were relatively low, their contribution to the phenotype and nutritional quality of PCC could be neglected. However, in other plant materials which contain a much higher proportion of these anthocyanidins, much more samples with a wide range of contents need to be included, and further optimization of their model-building methods is needed.

There are still some parts of NIR spectroscopy detection technology that need to be improved, but with the development of algorithm, spectroscopy, and artificial intelligence, the predictive ability, accuracy, and operability of this technology will continue to be improved on the original basis. With its obvious time-saving, high throughput, and non-destructive advantages, NIR spectroscopy will certainly have a broader development prospect in the agricultural, food industry, and market inspection.

## 5. Conclusions

Cyanidin was the most abundant anthocyanidin in purple leaf Chinese cabbage, with an average content of 5741.20 mg/kg, accounting for 95.7% of the total anthocyanidins. The prediction models established using visible/NIR spectroscopy on the basis of PLSR after no scattering processing and first-order derivative pretreatment method were suitable and effective for accurate and fast quantification of cyanidin and total anthocyanidin contents in PCC. The result laid a foundation for the application of NIR, with its obvious timesaving, convenience, and organic solvents free advantages, in the fast prediction of anthocyanidins in vegetables and rapid screening of purple vegetable breeding materials.

## Figures and Tables

**Table 1:** Distribution of anthocyanidins contents in purple leaf Chinese cabbage (mg/kg).

Compound	Content Range	Average Content	Percentage of Total %
delphinidin	nd^1^~193.7	159.4	2.66
cyanindin	93.5~12,802.4	5741.2	95.71
pelargonidin	nd^1^~66.0	52.3	0.87
peonidin	nd^1^~63.0	45.4	0.76

nd^1^: not detected.

**Table 2:** Calibration equations of cyanidin content in purple leaf Chinese cabbage using different pretreatment models.

Wave Band	Spectral Pre-Treatment Model	RSQ ^1^	SEC ^2^	1-VR ^3^	SECV ^4^
400~1100 nm 1100~2498 nm	None (no derivative)	0.922	808.339	0.908	887.788
	SNV+Detrend (no derivative)	0.928	772.348	0.894	948.462
	SNV only (no derivative)	0.913	852.994	0.866	1063.419
	Detrend only (no derivative)	0.942	685.539	0.923	801.505
	Standard MSC (no derivative)	0.923	784.604	0.896	924.328
	Weighted MSC (no derivative)	0.937	748.514	0.908	909.465
	None (first-order derivative)	0.965	531.591	0.942	693.004
	SNV+Detrend (first-order derivative)	0.959	576.934	0.931	754.230
	SNV only (first-order derivative)	0.956	602.184	0.924	799.911
	Detrend only (first-order derivative)	0.955	592.659	0.941	684.969
	Standard MSC (first-order derivative)	0.955	603.501	0.924	796.853
	Weighted MSC (first-order derivative)	0.952	622.972	0.917	825.123

^1^ RSQ: external correlation coefficient; ^2^ SEC: standard error of calibration set; ^3^ 1-VR: internal cross-validation correlation coefficient; ^4^ SECV: standard error of cross-validation.

**Table 3:** Calibration equations of total anthocyanidins content in purple leaf Chinese cabbage using different pretreatment models.

Wave Band	Spectral Pre-Treatment Model	RSQ ^1^	SEC ^2^	1-VR ^3^	SECV ^4^
400~1100 nm 1100~2498 nm	None (no derivative)	0.925	801.928	0.911	881.019
	SNV+Detrend (no derivative)	0.929	773.686	0.896	950.028
	SNV only (no derivative)	0.915	854.883	0.869	1062.558
	Detrend only (no derivative)	0.939	710.771	0.916	846.407
	Standard MSC (no derivative)	0.924	787.616	0.898	926.614
	Weighted MSC (no derivative)	0.938	749.215	0.910	910.829
	None (first-order derivative)	0.966	532.072	0.944	685.994
	SNV+Detrend (first-order derivative)	0.959	576.751	0.932	751.574
	SNV only (first-order derivative)	0.956	602.713	0.925	802.668
	Detrend only (first-order derivative)	0.956	592.196	0.941	691.249
	Standard MSC (first-order derivative)	0.956	595.245	0.930	761.400
	Weighted MSC (first-order derivative)	0.953	620.968	0.920	819.108

^1^ RSQ: external correlation coefficient; ^2^ SEC: standard error of calibration set; ^3^ 1-VR: internal cross-validation correlation coefficient; ^4^ SECV: standard error of cross-validation.

## Data Availability

Data is contained within the article or supplementary material.

## Supplementary Materials

The following supporting information can be downloaded at: https://www.mdpi.com/article/10.3390/foods12091922/s1, Table S1: Contents of anthocyanidins in purple Chinese cabbage analyzed by high-performance liquid chromatography (mg/kg).
