# Supplementary material for: Prediction of Anthocyanidins Content in Purple Chinese Cabbage Based on Visible/Near Infrared Spectroscopy
Source: Foods. 2023 May 8;12(9):1922. doi: 10.3390/foods12091922 (PMC10178596; doi:10.3390/foods12091922)
Supplement: Supplementary file 1 [file foods-12-01922-s001.zip › foods-2328017-supplementary.pdf]

Table S1. Contents of anthocyanidins in purple Chinese cabbage analyzed by high-performance liquid chromatography (mg/kg)

No.	delphinidin	cyanindin	pelargonidin	peonidin	No.	delphinidin	cyanindin	pelargonidin	peonidin	No.	delphinidin	cyanindin	pelargonidin	peonidin
**1**	173.9	3698.3	54.5	46.0	**37**	179.3	4035.7	55.1	47.0	**73**	155.6	10600.6	54.7	52.4
**2**	171.2	5005.9	52.9	47.8	**38**	162.7	4385.8	54.4	43.6	**74**	164.8	10149.0	57.0	54.5
**3**	159.8	4542.4	49.1	45.0	**39**	167.5	6240.8	55.8	45.9	**75**	169.7	9711.2	53.8	57.7
**4**	150.3	4524.0	48.7	41.1	**40**	171.8	5947.6	56.7	46.8	**76**	158.8	10101.2	52.3	49.4
**5**	163.4	5417.6	49.9	42.8	**41**	170.6	6450.3	55.5	48.1	**77**	170.2	12656.1	57.1	57.5
**6**	173.2	7634.0	59.8	46.7	**42**	193.7	7279.4	61.9	54.1	**78**	183.6	12579.2	64.0	56.7
**7**	164.9	5861.8	55.4	45.9	**43**	181.4	7056.3	60.2	52.8	**79**	181.3	12368.4	61.8	55.4
**8**	149.3	4638.2	50.1	40.7	**44**	181.1	7746.5	62.3	51.9	**80**	185.1	11785.7	58.6	57.5
**9**	134.6	5056.7	43.1	36.3	**45**	167.7	7721.1	56.6	48.2	**81**	nd	373.7	nd	nd
**10**	151.8	847.0	46.2	35.9	**46**	168.5	6986.2	56.4	49.1	**82**	nd	149.7	nd	nd
**11**	165.4	1498.3	51.0	39.8	**47**	172.1	7197.5	56.7	47.2	**83**	nd	93.5	nd	nd
**12**	149.6	792.7	44.3	33.9	**48**	174.0	5756.0	59.2	48.8	**84**	187.4	5103.6	60.4	52.0
**13**	165.2	1788.9	49.2	40.0	**49**	150.7	3437.1	47.2	42.6	**85**	182.9	8246.2	65.2	54.5
**14**	146.7	2247.5	46.2	36.1	**50**	164.2	4211.3	52.6	46.1	**86**	166.9	6705.6	59.4	48.6
**15**	140.9	1430.3	43.6	34.2	**51**	189.7	3737.0	60.2	52.1	**87**	150.7	819.8	45.2	34.9
**16**	177.4	2334.5	55.4	44.5	**52**	154.0	2375.9	47.7	40.3	**88**	175.7	5610.4	56.9	50.1
**17**	166.9	3305.5	52.6	42.5	**53**	158.0	3517.8	47.8	42.5	**89**	nd	419.9	nd	nd
**18**	172.2	2733.0	54.3	44.5	**54**	168.1	3391.4	52.7	46.4	**90**	158.5	5672.9	51.8	51.8
**19**	189.5	5460.9	62.4	54.4	**55**	169.1	2667.0	52.9	43.9	**91**	145.8	4482.9	47.7	47.1
**20**	164.7	3882.3	52.4	46.4	**56**	141.2	3494.8	44.7	39.2	**92**	153.9	3928.3	44.5	35.8
**21**	152.1	4930.9	50.1	45.7	**57**	137.6	2859.6	44.4	38.3	**93**	159.7	4824.4	59.1	49.0
**22**	165.7	6118.2	54.7	48.8	**58**	147.2	3787.7	45.7	40.3	**94**	150.6	4626.4	58.7	41.6
**23**	183.5	5333.7	58.9	49.6	**59**	147.9	7160.7	52.6	48.0	**95**	173.6	7833.9	53.7	47.7
**24**	187.3	7174.0	66.0	53.4	**60**	163.0	10658.0	58.2	55.9	**96**	150.9	1132.3	53.5	34.1
**25**	165.2	6582.6	54.6	49.1	**61**	157.7	5100.1	52.3	46.2	**97**	177.5	2234.2	57.4	45.6
**26**	156.5	4343.1	51.0	44.6	**62**	158.9	7828.1	55.9	49.6	**98**	157.2	2831.8	54.2	44.2
**27**	166.1	5067.9	52.7	45.8	**63**	156.6	9245.0	57.1	52.3	**99**	149.8	3961.5	46.5	42.3

**28**	185.8	5102.5	59.1	51.4	**64**	161.0	4813.1	54.9	44.3	**100**	171.1	7865.3	63.3	55.6
**29**	178.5	6412.4	58.0	51.3	**65**	171.2	6068.5	55.5	49.8	**101**	148.5	7081.9	56.8	59.1
**30**	188.8	9625.5	63.6	57.5	**66**	175.6	12802.4	62.0	63.0	**102**	192.7	3796.2	63.2	57.1
**31**	154.5	4460.9	48.8	41.0	**67**	149.1	8369.6	51.1	51.2	**103**	159.1	1767.3	42.9	50.9
**32**	170.9	7017.2	59.7	46.0	**68**	169.1	6745.0	58.7	50.7	**104**	169.5	8336.6	51.3	61.2
**33**	149.6	4031.2	46.6	42.4	**69**	157.8	9136.8	55.8	47.3	**105**	159.6	316.7	58.3	54.7
**34**	143.8	4454.9	46.3	40.6	**70**	141.8	7897.9	49.8	46.1	**106**	169.9	6805.0	49.5	38.6
**35**	178.1	6792.7	61.8	49.7	**71**	152.1	6231.8	49.8	45.7					
**36**	169.1	5454.1	55.0	44.9	**72**	144.4	5178.2	49.7	41.9					

No. 1-86: the calibration set samples; No. 87-106: the validation set samples.

nd: not detedcted.
